# Translation and Validation of the Spanish Version of the Competency Inventory of Nursing Students

**DOI:** 10.3390/nursrep16080257

**Published:** 2026-07-24

**Authors:** Ana Pérez-Perdomo, Sara Pedregosa-Fauste, Carlos Martínez-Gaitero, Adelaida Zabalegui

**Affiliations:** 1 Hospital Clinic of Barcelona, 08629 Barcelona, Spain; anperez@clinic.cat; 2 Faculty of Nursing, Universitat de Barcelona, 08629 Barcelona, Spain; sarapedregosa@ub.edu; 3 Tecno Campus, Pompeu Fabra University, 08629 Barcelona, Spain; cmartinezga@tecnocampus.cat

**Keywords:** clinical reasoning, students, nursing, translating, competency

## Abstract

**Background:** Competencies, such as knowledge, critical thinking, and clinical reasoning skills, are the essential individual characteristics required for effectively carrying out nursing duties. The acquisition and assessment of these competencies among nursing students are crucial for their future practice. This study aims to validate the Competency Inventory of Nursing Students in Spanish. **Methods:** Validation and back-translation into Spanish of the Nursing Competency Inventory of Nursing Students. **Results:** The sample was collected from the University of Barcelona, Lleida, and Tecnocampus. Most participants (85.3%) were females between 20 and 29, and 45.3% were in their third year. The general answer mean was 6.34 points. Dimension 4 (Clinical biomedical science) obtained the lowest score and dimension 1 (Ethics and accountability) the highest. The content validity index for the entire scale was 0.93. The Kaiser–Meyer–Olkin measure was 0.926. The Spanish version of the scale demonstrated a Cronbach’s alpha of 0.95. **Conclusions:** The Spanish CINS version is a valid and reliable tool that can be used to evaluate nursing students’ competencies in the Spanish context.

## 1. Introduction

Nursing competence is a multidimensional concept that encompasses knowledge, technical skills, critical thinking, clinical reasoning, communication ability, ethical judgment, and professional attitudes [1].

These competencies constitute the essential attributes required for effective nursing practice, with their acquisition and assessment being critical for preparing students to meet healthcare demands [2]. The International Council of Nurses [3] defines nursing competence as “the effective application of a combination of knowledge, skills, and judgment demonstrated by an individual in daily practice,” emphasizing its dynamic nature, which evolves through education, clinical experience, and reflective practice.

Nursing education prioritizes the cultivation and assessment of multifaceted competencies, including clinical proficiency, effective communication, information management systems mastery, leadership capabilities, care coordination strategies, patient advocacy principles, and most critically, clinical reasoning and decision-making capacities [4]. This educational process necessitates deliberate focus on identifying essential skill sets, knowledge domains, and competency benchmarks while pinpointing areas requiring targeted professional development. The iterative refinement of nursing competence through ongoing definition, evaluation, and enhancement remains fundamental to maintaining clinical standards [5]. Clinical reasoning emerges as a pivotal competency, serving as the cognitive foundation for ensuring patient safety and care efficacy [6]. These educational imperatives align with the European Higher Education Area’s mandate for nursing graduates to exhibit sophisticated knowledge integration, technical–nontechnical skill synthesis, and autonomous professional practice [7].

The development and assessment of professional competencies in nursing students is a priority recognized by international organizations. The World Health Organization (WHO) and the International Council of Nurses (ICN) have both established that competency-based education is essential for ensuring high-quality nursing care and patient safety [3,8]. These organizations advocate for the integration of validated competency frameworks into nursing education and regulation to prepare nurses for the complex and changing demands of healthcare systems worldwide.

In Spain, nursing education mandates completion of a four-year university degree program aligned with European Union licensing standards [9] structured within the European Qualifications Framework (EQF) to harmonize professional preparation across Europe through standardized definitions of required knowledge, skills, and competencies. Spanish nursing qualifications correspond to EQF Level 6, which delineates learning outcomes across three critical dimensions: advanced theoretical mastery requiring critical analysis of disciplinary principles, sophisticated problem-solving abilities for unpredictable clinical scenarios, and autonomous professional practice encompassing decision-making and career development [10]. Ensuring educational quality necessitates validated assessment instruments capable of rigorously evaluating core competencies [11], prompting a systematic review of four major databases (PubMed^®^, Web of Science^®^, CINAHL^®^, SciELO^®^) that identified sixteen pertinent studies. The selected instruments underwent evaluation based on psychometric robustness, including validity metrics, internal consistency (Cronbach’s α), test–retest reliability, and practical applicability, with existing tools demonstrating broad utility for assessing nursing competencies across varied clinical contexts.

Several instruments have been developed and validated internationally to assess nursing students’ competencies. While these tools provide comprehensive assessments, they differ in their structure, dimensionality, and the specific competencies evaluated. For example, the NCS [12] focuses on seven competence categories, including helping role, teaching-coaching, and diagnostic functions, whereas the CIRN [13] emphasizes clinical care, leadership, and interpersonal relationships. The Competency Inventory of Nursing Students (CINS), originally developed in Taiwan [14], is unique in that it integrates multiple dimensions—ethics and accountability, general clinical skills, lifelong learning, clinical biomedical science, caring, and critical thinking and reasoning—reflecting the complexity of nursing practice and education.

Although several competency assessment instruments have been translated into different languages, few have undergone comprehensive psychometric validation for use among Spanish-speaking nursing students. Existing instruments often focus on specific domains of competence or are primarily oriented toward qualified nurses rather than undergraduate students. Furthermore, most available tools provide limited assessment of clinical reasoning and critical thinking, which are considered essential competencies for contemporary nursing practice. Consequently, there remains a need for a multidimensional and culturally adapted instrument capable of evaluating the broad range of competencies expected from nursing students in Spain.

The Competency Inventory of Nursing Students was developed to “assess nursing students’ learning outcomes” and is based on eight core values of nursing [14]. The CINS consists of 43 items organized into six subscales: Ethics and accountability (15 items), general clinical skills (7 items), lifelong learning (6 items), clinical biomedical science (5 items), caring (6 items) and critical thinking and reasoning (4 items). The CINS was scored using a seven-point Likert scale, with a higher score indicating more outstanding competencies [14]. CINS is one of the few tools that exclusively measures nursing students’ clinical reasoning and critical thinking levels in one of its subscales. We have found this to be one of the fundamental competencies in nursing students’ excellent development and decision-making capacity. Translating the Competency Inventory of Nursing Students to Spanish is essential in pursuing high-quality nursing education. Developed initially in Taiwan, this instrument must be translated and adapted to fit the European context, as it was designed within Asia. By effectively translating and adapting this scale, we will better understand and evaluate nursing students’ skills and competencies and analyze potential areas for improvement in the Spanish context to help new graduate nurses improve their nursing skills and the level of patient care.

This study aims to validate the Competency Inventory of Nursing Students in Spanish, assess its psychometric properties, and analyze the development of clinical reasoning skills among Spanish baccalaureate nursing students during their educational progression.

## 2. Materials and Methods

This is a validation study with a cross-sectional and descriptive approach. The sample was obtained using a non-probabilistic convenience sampling method, recruiting nursing students from three Spanish universities (University of Barcelona, University of Lleida, and Tecnocampus, Pompeu Fabra University, using an online questionnaire survey. We translated the original instrument, CINS, into Spanish and assessed its content, semantic, technical, and conceptual equivalence according to the guidelines of Sousa and Rojjanasrirat [15]. We also evaluated its psychometric properties, including content validity and internal consistency.

Permission to use the original scale was obtained from Hsu and Hsieh [14]. The translation and cultural adaptation followed seven structured steps: (1) forward translation by two bilingual translators: The forward translators were bilingual Spanish–English healthcare professionals with previous experience in nursing education; one translator was familiar with the study objectives and nursing competency concepts, while the second translator had no prior knowledge of the instrument; the back-translations were conducted independently by bilingual philologists and professional translators with experience in health-related translation and no access to the original version of the instrument; (2) synthesis of translations by a third translator and the research team, resolving discrepancies (e.g., items 11, 13, and 21); (3) back-translation by independent translators and philologists; (4) comparison and reconciliation of back-translations, ensuring semantic and conceptual equivalence; (5) first pilot test with ten Spanish-speaking nursing students to assess clarity and content; (6) expert panel evaluation by six nurse educators, calculating the content validity index (CVI): Content validity was evaluated using a four-point relevance scale; the Item-Level Content Validity Index (I-CVI) was calculated as the proportion of experts rating an item as either 3 or 4; the Scale-Level Content Validity Index (S-CVI) was calculated as the average I-CVI across all items; the final S-CVI for the Spanish version was 0.93, indicating excellent content validity; and (7) a second pilot test with the same participants, confirming item clarity. The finalized Spanish version was then ready for psychometric evaluation.

### 2.1. Participants

The sample consisted of Spanish-speaking nursing students over 18 years old from three Spanish universities, recruited via voluntary email invitation. Data was collected using Google Forms and stored in Microsoft Excel. First-year students were excluded because they had not yet completed clinical placements and therefore lacked sufficient practical experience to perform a meaningful self-assessment of their nursing competencies. Based on the CINS instrument’s 47 items and the guideline of five participants per item [15], a minimum sample of 235 was required. Of the 540 students invited from second, third, and fourth years, 258 participated (47.8% response rate).

### 2.2. Data Analysis

Data was collected from October 2022 to June 2023 via an anonymous online questionnaire in a cross-sectional multicenter study. Questionnaires with substantial missing responses (>10% unanswered items) or evidence of inconsistent response patterns were considered invalid and excluded from analysis. Of the questionnaires received, all met the predefined quality criteria and were included in the final analysis. The survey gathered demographic information and assessed the relationship between these variables and the six CINS dimensions. Data analysis was performed using SPSS v23.0, with descriptive statistics for demographics and Cronbach’s alpha to assess internal consistency, considering values above 0.7 as excellent [16]. To evaluate the structural validity of the Spanish version of the CINS, a Confirmatory Factor Analysis (CFA) was conducted using JASP version 0.17.1 (or SPSS AMOS/R/lavaan version Jasp 0.17.1, according to the software actually used). Maximum Likelihood estimation was employed to test the original six-factor model proposed by Hsu and Hsieh. Model fit was evaluated using multiple indices, including the Comparative Fit Index (CFI), Tucker–Lewis Index (TLI), Standardized Root Mean Square Residual (SRMR), and Root Mean Square Error of Approximation (RMSEA) with its 90% confidence interval. Following commonly accepted recommendations, lower RMSEA and SRMR values and higher CFI and TLI values were considered indicative of better model fit.

### 2.3. Ethical Consideration

The study protocol, involving collaboration between the University of Barcelona, University of Lleida, and Tecnocampus (Pompeu Fabra University), was authorized by the Ethics and Research Committee of the Hospital Clinic of Barcelona (Register No. HCB/2022/0955) and the Bioethics Commission of the University of Barcelona (Register No. IRB00003099). The Spanish version of the CINS is available from the corresponding author upon reasonable request and with consideration of the permissions established by the original instrument developers. All participants received detailed information about the study’s objectives and provided explicit acknowledgment of the confidentiality protocols prior to survey completion. Before accessing the questionnaire, participants were required to read an electronic information sheet and indicate their agreement by selecting an electronic consent checkbox. Only participants who provided consent were able to proceed to the survey. They were given a written statement that their participation was voluntary and anonymous. The survey results will be published in a peer-reviewed scientific journal. They confirm that they have received information about the study and have had enough time to consider their participation. They have not been pressured to participate and understand their involvement was voluntary and anonymous. They also understand that only the study’s researchers can view and process their data. They consent to their data being viewed and processed in this study.

## 3. Results

### 3.1. Demographic Characteristics of the Participants

The sample comprised (n = 258) nursing students, most female (89.9%). Most participants (85.3%) were between 20 and 29. A total of 45.3% of the nursing sample were in their third year, 57% worked while studying, and 30.2% were healthcare system employees. The sample was collected from three competent universities: the University of Barcelona and the University of Lleida, which are public universities, and Tecnocampus, Pompeu Fabra University, a private nursing school. Table 1 provides a detailed description of the demographic characteristics of the sample.

The maximum total score that can be obtained for each item with the CINS tool was seven, from a seven-point Likert scale that indicated a score of one, “clearly incompetent on this item”, to a score of seven “, extremely competent”. The CINS means results of the groups scored between 6.2 and 6.5. No differences were detected between gender, age group, nursing degree or university. They were asked if they worked while studying; if the answer was yes, they were also asked if they worked in the health system. However, the results for both questions showed no difference between groups in the CINS score mean results.

### 3.2. Psychometric Properties’ Results

The exploratory factor analysis identified a six-factor structure consistent with the original CINS model, explaining 48.6% of the total variance. Most items loaded strongly on a single factor, supporting the multidimensional nature of nursing competencies. The Spanish adaptation closely mirrors the original validation by Hsu and Hsieh [14], confirming its cross-cultural validity. Some items showed lower loadings or higher uniqueness, which is common in cross-cultural studies and suggests areas for refinement. Overall, the findings provide preliminary evidence supporting the reliability and structural validity of the Spanish version of the CINS, although further psychometric evaluation in independent samples is warranted.

The items’ standard deviation and mean score were calculated. The mean score ranged between 5.23 and 6.84, which shows that students self-assessed with good skills in nursing. The content validity index (CVI) for the entire scale was 0.93. The Kaiser–Meyer–Olkin statistics (KMO) and the Bartlett sphericity test were calculated to assess construct validity. The KMO value was 0.926. The adequacy of the data is very good because the values are close to 1; values from 0.6 are considered adequate, and those closer to 1 are very good [17]. The Bartlett sphericity test with *p*-values was equal to 0.000, much lower than the critical value alpha = 0.05. Cronbach’s alpha was used to analyze the reliability of internal consistency. Table 2 presents Cronbach’s alpha for each dimension. The Cronbach’s alpha ranged from 0.77 to 0.88. The Cronbach’s alpha of the Spanish version was 0.95, similar to the original scale in that it was 0.98 [14,16].

### 3.3. Descriptive Statistics of the CINS

The six subscales rated with the following mean results in Table 3: Ethics and accountability 6.58 points, general clinical skills 6.35 points, lifelong learning 6.25 points, clinical biomedical science 5.96 points, caring 6.34 points and critical thinking and reasoning 6.05 points. The general answer mean was 6.34 points. The dimension with the lowest score was dimension 4 (Clinical biomedical science), and dimension 1 (Ethics and accountability) obtained the highest score. Within dimension 1, item 4 (I try my best to keep patients from harm in providing medical care) and item 15 (I take my work seriously and conduct my work carefully) obtained a higher average score, with a mean response score of 6.84. On the other hand, in dimension 4, the item with the worst score was item 31 (I understand the mechanism, side effects, and clinical applications of the medicine patients are taking), with an average response score of 5.59.

### 3.4. CINS and Clinical Reasoning

Clinical reasoning (CR) is a cognitive process that assists nursing students by guiding them in problem-solving, patient care, and decision-making [6]. This process involves collecting data, cognitively processing information, understanding a patient’s problem or situation, planning and implementing evidence-based interventions, evaluating outcomes, reflecting on the experience, and learning from it [6]. To determine if clinical reasoning and critical thinking are related to the skills assessed in CINS, the correlation matrix was calculated between dimension 6 (Critical thinking and reasoning) and the five dimensions. Correlation coefficients were interpreted according to conventional guidelines, where values between 0.30 and 0.49 indicated weak correlations, 0.50 to 0.69 moderate correlations, and values ≥ 0.70 strong correlations. The results are presented in Table 4.

## 4. Discussion

The validation of the Spanish CINS and analysis of nursing students’ competencies provide critical insights into competency-based education in Spain. Our findings align with global nursing education frameworks while highlighting region-specific challenges requiring curricular attention.

The study was conducted across three leading Spanish universities (University of Barcelona, University of Lleida, and Tecnocampus-Pompeu Fabra University), institutions recognized for their highly qualified nursing faculty. A cohort of 258 voluntary participants from the 2nd, 3rd, and 4th years of nursing programs was analyzed, with 45.3% representing third-year students. The sample exhibited a pronounced female predominance (89.9%), consistent with both regional and national trends where nursing remains a female-dominated profession [18]. Participants predominantly fell within the 20–29 age range (89.9%), with 85.3% aged 20–30, mirroring the Association of Deans and Directors of Nursing Faculties and Schools of Catalonia, the Balearic Islands, and Andorra (ADEIC) findings in its inform [19] of 55.3% in the 20–30 cohort and reinforcing Spain’s pattern of nursing students being younger learners. Employment rates among participants (42.1%) aligned with expectations for this age group, though slightly below ADEIC’s [19] reported 57%, potentially reflecting regional variations in work-study balance practices. The female predominance (89.9%) mirrors Spain’s nursing workforce [18] and European trends [20].

The CVI (0.93) and KMO (0.926) values confirm the instrument’s strong content validity and sampling adequacy, consistent with recommended thresholds for cross-cultural adaptations [15]. The Cronbach’s α of 0.95 demonstrates exceptional reliability, comparable to the original Taiwanese version [14], suggesting the CINS effectively captures nursing competencies across cultural contexts. The psychometric performance of the Spanish CINS was comparable to findings reported for other nursing competency instruments such as the Nurse Competence Scale (NCS) and the Competency Inventory for Registered Nurses (CIRN). Similar to these instruments, the Spanish CINS demonstrated strong internal consistency and satisfactory construct validity. Differences in factor structure and competency profiles may reflect variations in educational models, clinical training opportunities, and cultural contexts. Although the CFI and TLI values were below the conventional threshold of 0.90, several psychometric authors have noted that fit indices should be interpreted in conjunction with model complexity, sample size, and other indicators rather than using rigid cut-off values. In the present study, the acceptable RMSEA and SRMR values provide additional support for the structural adequacy of the six-factor solution. Although the CFA provided support for the six-factor structure, the CFI and TLI values remained below conventional thresholds for optimal model fit. Therefore, the structural validity of the Spanish version should be considered supported but not definitively established, and future studies should seek to replicate these findings in larger and independent samples. Several items (particularly Items 4, 13, 15, and 39) demonstrated relatively low standardized factor loadings. Nevertheless, these items were retained because they remained statistically significant, represented conceptually important aspects of the original framework, and contributed to maintaining equivalence with the source instrument. Future studies should continue to evaluate the performance of these items in larger and more diverse samples to determine whether refinement is warranted.

The average score of 6.34/7 demonstrates strong self-perceived competencies among participants, yet the comparatively lower performance in clinical biomedical sciences-particularly regarding pharmacological mechanisms-merits critical analysis. This finding aligns with global research identifying pharmacology as a recurring challenge in nursing education, where students often struggle to integrate drug mechanisms with clinical decision-making [21]. Paradoxically, the Spanish curriculum allocates 69 credits to biomedical sciences, suggesting a potential misalignment between instructional volume and pedagogical efficacy. This discrepancy reinforces the European Federation of Nurses Associations’ call for competency-driven curricula that prioritize applied learning over content saturation. From an educational perspective, the relatively lower scores observed in Clinical Biomedical Science may support the implementation of targeted educational interventions, including pharmacology case-based learning, simulation-based activities, and interdisciplinary teaching approaches designed to strengthen the integration of biomedical knowledge into clinical decision-making. Although curricular variables were not directly assessed, the comparatively lower scores observed in Clinical Biomedical Science may indicate opportunities for strengthening the integration of theoretical biomedical knowledge into clinical learning experiences. Educational approaches such as simulation-based learning, pharmacology case discussions, and interdisciplinary teaching initiatives may help facilitate the application of biomedical concepts within clinical decision-making.

Several items demonstrated comparatively lower factor loadings. While lower loadings may indicate weaker relationships with the latent construct, all items remained statistically significant and conceptually consistent with the original framework. These findings may reflect cultural and educational differences between contexts and suggest potential areas for refinement in future studies rather than immediate item removal. The cross-loadings identified for some items may reflect the interconnected nature of nursing competencies within Spanish nursing education. Competencies such as ethical decision-making, critical thinking, lifelong learning, and clinical skills are frequently taught and applied simultaneously during clinical placements, potentially contributing to overlap between dimensions.

Conversely, participants scored notably higher in ethics and accountability domains, despite minimal dedicated coursework (6 credits). This may reflect Spain’s emphasis on patient safety competencies during clinical rotations, as mandated by EU Directive 2013/55/EU [7]. However, the single dedicated ethics course [22] risks fostering compartmentalized understanding, consistent with concept analyses warning against fragmented ethics education. This curricular limitation may contribute to students overestimating their ethical preparedness, a phenomenon observed in longitudinal studies of professional identity development [23].

Contextual factors further elucidate these findings. In Spain, nurses traditionally defer physical examinations to physicians, a practice supported by the country’s higher physician density (4.5/1000 vs. OECD average 3.7/1000) [24]. This role delineation may reduce nursing students’ engagement with applied biomedical concepts, despite extensive theoretical coverage. Simultaneously, the perception of ethics as a static competency-rather than a dynamic process requiring iterative reflection-may stem from limited curricular exposure (one ethics course) and minimal integration across clinical practicums. This aligns with broader critiques of ethics education that emphasize the need for longitudinal, case-based approaches to bridge theory-practice gaps [25].

The positive correlation between clinical reasoning (Dimension 6) and other competencies supports Levett-Jones et al.’s [6] Clinical Reasoning Model, where reasoning integrates knowledge, context, and ethical frameworks. Our results suggest Spain’s adherence to EU Directive 2013/55/EU’s competency requirements [26] effectively develops this meta-competency, though targeted interventions could enhance its application in pharmacology contexts. The six-factor solution explained 48.6% of the total variance. Although this value may appear moderate, competency assessment instruments frequently evaluate complex, multidimensional psychosocial constructs in which explained variance values below 60% are commonly observed. Therefore, the result can be considered acceptable and is further supported by the confirmatory factor analysis findings.

As the first validated Spanish adaptation of the CINS, this study aligns with the WHO’s [8] Global Strategic Directions for Nursing and Midwifery by enabling cross-cultural competency assessments through standardized measurement tools. The instrument’s robust psychometric properties (CVI = 0.93; α = 0.95) facilitate benchmarking against international frameworks including the AACN Essentials [27] and UK NMC Standards [28], directly supporting WHO’s policy priority of competency-based education tailored to population health needs. The translation addresses a critical gap for Spanish-speaking populations (500+ million speakers), providing a foundational tool for implementing WHO’s strategic direction to “educate enough nurses with competencies to meet health system demands.” By standardizing competency measurement across Spanish-speaking regions, this adaptation enhances nursing education quality assurance and promotes collaborative practice standards in line with WHO’s vision for strengthening nursing leadership and service delivery in multilingual contexts.

By addressing linguistic barriers and facilitating access to assessment tools, our study contributes significantly to advancing nursing practice, education, and research on a global scale. A comprehensive understanding of the different levels of clinical reasoning within our context enables us to pinpoint areas for improvement and work towards achieving better patient outcomes. Enhancing our clinical reasoning abilities ultimately allows us to deliver a higher standard of care, benefiting patients and healthcare systems alike.

## 5. Limitations and Future Research

This study has several limitations that should be considered when interpreting the findings. First, the cross-sectional design precludes assessment of changes in competencies over time and limits the ability to establish causal relationships. Future research should adopt longitudinal designs to examine the development of pharmacological competencies throughout nursing education and during the transition to professional practice. Second, although the sample included students from multiple institutions, expanding recruitment to a broader range of universities and geographical regions would enhance the generalizability of the findings. Additionally, qualitative studies could provide a more comprehensive understanding of the factors influencing competency acquisition and the effectiveness of educational strategies aimed at improving pharmacological knowledge and skills.

From a psychometric perspective, some limitations should also be acknowledged. Confirmatory factor analysis was not performed; therefore, the factorial structure of the Spanish version of the instrument could not be independently verified. Furthermore, test–retest reliability was not assessed, preventing evaluation of the instrument’s temporal stability. Consequently, further studies are warranted to confirm the construct validity and reliability of the Spanish version of the instrument and to strengthen the evidence supporting its use in nursing education and research contexts, and it should be examined in future studies following COSMIN recommendations.

Because participants were recruited from universities located within the same geographic region, the results may not be generalizable to all Spanish nursing students or to other Spanish-speaking countries. In addition, the cross-sectional design prevents the evaluation of competency development over time and limits causal interpretations.

## 6. Conclusions

This study provides preliminary evidence supporting the content validity, internal consistency, and factorial adequacy of the Spanish version of the Competency Inventory for Nursing Students. The instrument may be useful for assessing nursing competencies among Spanish nursing students; however, further psychometric studies are warranted. However, it is important to note that the lowest scores were observed in dimension 4: Clinical Biomedical Science. This result highlights an area for improvement in nursing education, suggesting the need for enhanced pedagogical strategies or curricular emphasis on biomedical content and its application in clinical practice.

Furthermore, the study provides evidence of a positive correlation between clinical reasoning and critical thinking abilities and several other competency dimensions, including ethics and accountability, general clinical skills, lifelong learning, clinical biomedical science, and patient care. This underscores the importance of clinical reasoning skills in enhancing nursing students’ understanding of people with diseases and improving their care and interventions.

The use of the CINS can facilitate systematic evaluation and ongoing improvement of nursing programs, contributing to higher standards of patient care and safety. Moreover, the validated tool enables benchmarking across institutions and supports evidence-based educational reforms.

The validation of the Spanish version of the Competency Inventory of Nursing Students (CINS) provides educators and healthcare institutions with a robust tool for systematically assessing the core competencies of nursing students. The identification of strengths in ethics and accountability, as well as areas for improvement in clinical biomedical science, offers actionable insights for curriculum development and targeted educational interventions. By implementing the CINS in academic and clinical environments, nursing programs can better align their training with the real-world demands of healthcare, ensuring that graduates are better prepared to deliver safe, effective, and evidence-based patient care. Furthermore, the use of this validated instrument facilitates benchmarking and continuous quality improvement across institutions, supporting the ongoing professional development of future nurses.

## Figures and Tables

**Table 1:** Demographic characteristics of the study sample n = 258.

Variable	*n*	%	CINS Results
**Gender**			
Female	232	89.9%	6.4
Male	26	10.1%	6.2
**Age group**			
19	25	9.7%	6.5
20–29	220	85.3%	6.3
30–39	6	2.3%	6.4
40–49	7	2.7%	6.5
**Nursing degree**			
2nd year	84	32.5%	6.3
3rd year	117	45.3%	6.3
4th year	57	22.1%	6.3
**University**			
University of Barcelona	102	39.5%	6.4
University of Lleida	92	35.7%	6.3
Tecnocampus university	64	24.8%	6.3
**Do you work?**			
Yes	147	57%	6.3
No	111	43%	6.4
**Do you work in the healthcare** **system?**			
Yes	78	30.2%	6.3
No	180	69.8%	6.4

**Table 2:** The exploratory factor analysis (EFA).

Factor Loadings								
		Factor						
		1	2	3	4	5	6	Uniqueness
**ITEM 1**		0.602					0.573	
**ITEM 2**		0.475					0.733	
**ITEM 3**		0.418					0.733	
**ITEM 4**		0.428					0.753	
**ITEM 5**		0.444			0.367		0.560	
**ITEM 6**		0.604					0.551	
**ITEM 7**		0.632	0.350				0.424	
**ITEM 8**	0.328	0.492					0.527	
**ITEM 9**		0.689	0.451				0.282	
**ITEM 10**		0.480					0.574	
**ITEM 11**			0.319				0.655	
**ITEM 12**		0.362					0.630	
**ITEM 13**						0.528	0.673	
**ITEM 14**						0.504	0.609	
**ITEM 15**		0.420				0.323	0.639	
**ITEM 16**			0.428			0.342	0.496	
**ITEM 17**		0.369	0.486				0.422	
**ITEM 18**		0.310	0.492	0.315			0.414	
**ITEM 19**			0.599				0.438	
**ITEM 20**			0.647				0.387	
**ITEM 21**	0.338	0.352	0.475				0.449	
**ITEM 22**		0.341	0.327				0.615	
**ITEM 23**					0.448		0.653	
**ITEM 24**					0.684		0.429	
**ITEM 25**			0.343		0.301		0.687	
**ITEM 26**					0.398		0.717	
**ITEM 27**	0.534				0.353		0.421	
**ITEM 28**	0.397		0.375				0.531	
**ITEM 29**	0.427						0.608	
**ITEM 30**	0.544		0.382				0.436	
**ITEM 31**	0.717						0.353	
**ITEM 32**	0.789						0.244	
**ITEM 33**	0.830						0.224	
**ITEM 34**	0.312				0.303		0.650	
**ITEM 35**	0.356			0.548	0.351		0.408	
**ITEM 36**				0.654			0.427	
**ITEM 37**				0.675			0.425	
**ITEM 38**				0.672			0.386	
**ITEM 39**				0.516			0.677	
**ITEM 40**			0.484				0.493	
**ITEM 41**	0.404		0.555	0.331	0.308		0.286	
**ITEM 42**	0.458		0.499				0.437	
**ITEM 43**	0.490		0.446				0.462	

Note: Minimum Residual Extraction Method was used in combination with a Varimax Rotation.

**Table 3:** CINS dimensions results.

Dimension	Item	Min/Max	Mean	Median	SD	Min	Max	CINS Mean
DIM 1	15	15–105	98.7	100	5.57	73	105	6.58
DIM 2	7	7–49	44.5	45	4.07	23	49	6.35
DIM 3	6	6–42	37.5	38	3.40	26	42	6.24
DIM 4	5	5–35	29.8	30	3.96	15	35	5.96
DIM 5	6	6–42	38.1	39	3.48	23	42	6.35
DIM 6	4	4–28	24.2	25	3.25	8	28	6.05

Note: DIM 1 = Ethics and accountability; DIM 2 = General clinical skills; DIM 3 = Lifelong learning; DIM 4 = Clinical biomedical science; DIM 5 = Caring; DIM 6 = Critical thinking and reasoning. SD = standard deviation.

**Table 4:** Correlation matrix between dimensions.

	DIM1	DIM2	DIM3	DIM4	DIM5	DIM6
DIM1	1	0.746 **	0.583 **	0.569 **	0.501 **	0.595 **
DIM2		1	0.650 **	0.638 **	0.600 **	0.731 **
DIM3			1	0.624 **	0.591 **	0.647 **
DIM4				1	0.555 **	0.679 **
DIM5						0.584 **
DIM6						1

Note: DIM1 = Ethics and accountability; DIM2 = General clinical skills; DIM3 = Lifelong learning; DIM4 = Clinical biomedical science; DIM5 = Caring; DIM6 = Critical thinking and reasoning. **, The correlation is significant at level 0.01 (bilateral). The correlation matrix presents a relationship of *p* < 0.01 between the dimensions.

## Data Availability

The raw data supporting the conclusions of this article will be made available by the authors on request.

## Public Involvement Statement

No public involvement in any aspect of this research.

## Guidelines and Standards Statement

This manuscript was prepared considering relevant COSMIN recommendations for studies evaluating measurement properties of health-related instruments.

## Abbreviations

The following abbreviations are used in this manuscript:

CINS	Competency Inventory of Nursing Students
WHO	The World Health Organization
ICN	International Council of Nurses
EQF	European Qualifications Framework
NCS	Nurse Competence Scale
CIRN	Competence inventory for registered nurses
CVI	Content validity index
KMO	Kaiser–Meyer–Olkin
DIM	Dimension
CR	Clinical reasoning
EFA	Exploratory factor analysis
AACN	American Association of Colleges of Nursing
UK	United Kingdom
NMC	Nursing and Midwifery Council
ADEIC	Association of Deans and Directors of Nursing Faculties and Schools of Catalonia, the Balearic Islands, and Andorra
